# Effects of α-terpineol and 1,8-cineole from *Cinnamomum longepaniculatum* on serum inflammatory cytokine factor and cecal microbial diversity of growing meat rabbits

**DOI:** 10.3389/fvets.2026.1815444

**Published:** 2026-06-29

**Authors:** Li-Tao Che, Rui-Xin Zhou, Ahmed H. El-Sappah, Ahmed S. Eldomiaty, Amr M. Atif, Yong-Jun Ren, Qi-Fan Wu, Ya-Ru Yang, Guang-Hua Wang, Hong-Li Liu, Qin Wei

**Affiliations:** 1 Faculty of Agriculture, Forestry and Food Engineering, Yibin University, Yibin, China; 2 Sichuan Province Engineering Technology Research Center of Oil Cinnamon, Yibin, China; 3 Department of Genetics, Faculty of Agriculture, Zagazig University, Zagazig, Egypt; 4 Department of Microbiology, Faculty of Agriculture, Zagazig University, Zagazig, Egypt; 5 Sichuan Animal Science Academy, Chengdu, China; 6 Animal Breeding and Genetics Key Laboratory of Sichuan Province, Chengdu, China; 7 Kunming New Hope Agricultural Technology Co., Ltd, Kunming, China

**Keywords:** 1,8-cineole, cecal microbial diversity, *Cinnamomum longepaniculatum* essential oils, inflammatory cytokine factor, α-terpineol

## Abstract

**Introduction:**

*Cinnamomum longepaniculatum* essential oils have complex components with distinct properties. 1,8-cineole and α-terpineol are its main active components, while other components like safrole and camphor are toxic. To avoid interference from other components, and given the few studies on CNI and TEP in meat rabbit production, this study selected them to explore their application effects and mechanisms in meat rabbit production.

**Aims and methods:**

The effects on blood inflammatory cytokines and cecal microbial diversity in meat rabbits of α-terpineol, 1,8-cineole, and a combination of the two compounds from *Cinnamomum longepaniculatum* were investigated in this study. 160 New Zealand White rabbits that had just been weaned and were 35 days old were divided into five groups: control, antibiotic (enramycin 20 mg/kg + chlortetracycline 50 mg/kg), α-terpineol (80 mg/kg), 1,8-cineole (80 mg/kg), and combination α-terpineol + 1,8-cineole (40 mg/kg each). Following a 5-day adaptation period, the trial continued for an additional 42 days.

**Results:**

All treatment groups showed significantly lower levels of D-lactic acid, diamine oxidase, IL-1β, and TNF-α than the control. The anti-inflammatory effects of α-terpineol and 1,8-cineole were comparable to those of antibiotics when used independently. Clostridia abundance decreased significantly in the cecal microbiota. Actinobacteriota were enriched after different treatments, while Deferribacterota showed a remarkably lower level in treatment groups than in the control.

**Conclusion:**

Meat rabbits that were given 80 mg/kg of either α-terpineol or 1,8-cineole showed an improvement in blood inflammatory markers and cecal microbiota. Compared to the combination therapy, supplementation with 80 mg/kg of α-terpineol ingredient had better results.

## 1 Introduction

The worldwide initiative to limit and eradicate antibiotic growth promoters (AGPs) in livestock production has generated an immediate and essential demand for safe, effective, and scientifically substantiated alternatives that can sustain animal health, welfare, and productivity (1, 2). In intensive production systems, two closely connected biological processes—systemic inflammation and intestinal dysbiosis—are key factors influencing growth efficiency and disease susceptibility (3). The compromise of the intestinal epithelial barrier, commonly referred to as “leaky gut,” enables the translocation of luminal pathogens, microbial endotoxins such as lipopolysaccharide (LPS), and bacterial metabolites, including D-lactate, into the systemic circulation (4). This translocation triggers a robust inflammatory cascade, characterized by increased production of proinflammatory cytokines, including tumor necrosis factor-alpha (TNF-α) and interleukin-1 beta (IL-1β) (5, 6). This persistent, low-level inflammation not only diverts energy and resources from growth but also heightens susceptibility to gastrointestinal and systemic infections. The inflammatory environment significantly modifies the composition and functionality of the gut microbiota, diminishing its diversity and stability, thereby exacerbating intestinal dysfunction (7). Consequently, the advancement of next-generation dietary methods must employ a comprehensive strategy that concurrently focuses on improving intestinal barrier integrity, controlling systemic immune responses, and cultivating a robust and beneficial gut microbial community (8). Essential oils (EOs) derived from plants have emerged as promising alternatives to address this complex issue (9). Their extensive range of bioactivities, including antibacterial, anti-inflammatory, antioxidant, and immunomodulatory properties, provides a comprehensive approach to gut health management (10). Importantly, in contrast to traditional antibiotics that often target specific molecular sites in bacteria, EOs produce pleiotropic effects (11). They can damage microbial cell membranes, neutralize reactive oxygen species, and regulate host cell signaling pathways, including nuclear factor-kappa B (NF-κB), nuclear factor erythroid 2-related factor 2 (Nrf2), and mitogen-activated protein kinase (MAPK) cascades (12, 13). This multi-modal mechanism of action diminishes the selective pressure for antimicrobial resistance while providing broader physiological advantages. Nonetheless, a significant impediment to their standardized and reasonable use is their inherent phytochemical complexity. The biological efficacy of an essential oil is highly variable and depends on its chemotype, which is influenced by factors such as plant genetics, geographical origin, harvest period, and extraction method (14). This diversity generates significant ambiguity in product standardization, dosage assessment, and mechanistic interpretation, underscoring the urgent need to examine well-defined, purified bioactive components rather than complex crude combinations (15). *Cinnamomum longepaniculatum* (Gamble) N.Chao ex H.W.Li, an aromatic tree native to China, is a significant source of a terpenoid-rich essential oil (CLEO) with proven promise as a functional feed additive (16). The phytochemical analysis of CLEO has identified 1,8-cineole (eucalyptol) and α-terpineol as its primary monoterpenoids. Analyses demonstrate that the levels of these chemicals are directly correlated with the oil's antioxidant capacity (17). The functional importance of these components is further corroborated by evidence indicating their direct role in the oil's antibacterial efficacy (18). This study jointly identifies 1,8-cineole and α-terpineol as the principal bioactive constituents responsible for CLEO's essential functional features. An expanding collection of *in vitro* and preclinical research clarifies their unique but complementary processes. 1,8-Cineole is recognized as a potent anti-inflammatory compound that can help alleviate intestinal barrier dysfunction (19). Recent research indicates that it can inhibit the activation of the NF-κB and MAPK pathways, thereby downregulating the expression of cytokines such as TNF-α and IL-6 (20). Moreover, discoveries demonstrate that it directly augments the gene and protein expression of essential tight junction components, including claudin-1, occludin, and zonula occludens-1 (ZO-1), thereby strengthening the intestinal physical barrier (21). In contrast, α-terpineol exhibits superior direct antibacterial and antioxidant properties (22). Its lipophilic properties enable it to penetrate bacterial membranes and compromise their structural integrity, leading to cell lysis. Recent studies indicate that α-terpineol can activate the Nrf2 antioxidant pathway, thereby enhancing the production of endogenous cytoprotective enzymes such as superoxide dismutase (SOD) and glutathione peroxidase (GSH-Px), which protect enterocytes from oxidative stress-related damage and apoptosis (23). Notwithstanding these encouraging developments, significant and crucial information deficiencies remain, which the present work is explicitly intended to rectify. The current literature primarily emphasizes the effects of crude EOs or particular chemicals examined in isolation within rodent models or cell culture systems. There is a notable deficiency of controlled, comparative *in vivo* investigations in economically significant meat rabbits that directly assess the individual and combined effects of these isolated substances. The ecological impacts of 1,8-cineole and α-terpineol, individually and collectively, on the intricate community structure of gut microbiota in a physiologically relevant herbivore model are mainly unexplored. The effects of their simultaneous delivery on microbial diversity and essential bacterial taxa—whether synergistic, additive, or antagonistic—remain uncertain. Third, although their anti-inflammatory properties are established, a simultaneous analysis investigating their impact on validated serum markers of intestinal permeability (e.g., D-lactate and diamine oxidase) alongside deep sequencing-based profiling of the cecal microbiome in meat rabbits constitutes a novel and thorough method for evaluating gut health. This study was conducted to systematically examine the effects of dietary supplementation with purified 1,8-cineole and α-terpineol—administered both individually and in combination—on systemic inflammation and cecal microbial ecology in growing meat rabbits. The effectiveness of these phytogenic therapies was meticulously assessed in comparison to an unsupplemented negative control and a positive control group administered a standard antibiotic regimen of enramycin and chlortetracycline. This study seeks to provide a conclusive, comparative evaluation of the distinct and interaction roles of these two essential terpenoids in maintaining gut health homeostasis, utilizing serum biomarker analysis and high-throughput 16S rRNA gene sequencing. The results are anticipated to establish a solid scientific basis for the logical development of targeted, effective, and dependable phytogenic feed additives as sustainable substitutes for in-feed antibiotics.

## 2 Materials and methods

### 2.1 Ethics statement

All experimental techniques employed in this work received clearance from the Ethics Committee of Life Sciences at Yibin University (clearance Number: 2024031201, Date of Approval: March 12, 2024). All experimental procedures were performed in accordance with relevant institutional guidelines and regulations. Moreover, this study is reported in accordance with the ARRIVE guidelines.

### 2.2 Materials

1,8-Cineole (99.5%) and α-Terpineol (98.0%) are extracted, concentrated, and separated from the leaves of *Cinnamomum longepaniculatum* in Yibin, Sichuan Province, by Yibin Shiping Spice Co., Ltd., using steam distillation (patent numbers: ZL 2018 2 1792919.9; ZL 2018 2 1792407.2). The two EOs were then encapsulated with β-cyclodextrin into 10% feed additives by Chongqing Wedidit Pharmaceutical Co., Ltd, and stored for future use. The composition and content of the two EOs and the encapsulated essential oil feed additive product had been tested and qualified using gas chromatography. Robenidine hydrochloride (10%), Diclazuril (0.5%), enramycin (4%), and chlortetracycline hydrochloride (15%) were purchased from Sichuan Ruifangde Bio-Pharmaceutical Co., Ltd.

### 2.3 Experimental design

The experiment was conducted at the cooperative farm of the Sichuan Animal Science Academy (April 1–May 17, 2024).160 healthy 35-day-old weaned New Zealand rabbits (half male and half female) were randomly divided into five groups with eight replicates per group and four rabbits per replicate: control group (CON group), antibiotic group (ANT group),α-Terpineol group (TEP2 group), a 1,8-Cineole group (CNI group), and a combined essential oil group of 1,8-Cineole and α-Terpineol (TEP2+CNI group). The CON group was fed a basal diet. Based on China's pre-antibiotic ban, the Livestock and Poultry Drug Manual, and the actual conditions of rabbit production, the ANT group was supplemented with antibiotics (20 mg/kg enramycin and 50 mg/kg chlortetracycline) in the basal diet. The TEP2, CNI, and TEP2+CNI groups were supplemented with α-Terpineol (80 mg/kg), 1,8-Cineole (80 mg/kg), and a composite essential oil (α-Terpineol 40 mg/kg + 1,8-Cineole 40 mg/kg) in the basal diet, respectively. The stated doses of the additive for all experimental groups refer to the dose of the active ingredient. The doses of EOs components were selected based on previous studies (eg, 20–150 mg/kg), and 80 mg/kg was chosen as the median value (24, 25). The equal total dose in the combination group was designed to evaluate whether the composite essential oil produces additional benefits compared to each component alone at the same total supplementation level. All rabbits were fed the corresponding diets accordingly. The preparation period was 5 days, and the formal experiment period was 42 days.

### 2.4 Experimental diet

The basal diet (Table 1) was formulated according to the Nutrient Requirements of Meat Rabbits (NY/T 4049-2021) (26), in combination with the growth characteristics of New Zealand meat rabbits. Its composition and nutritional levels are presented in Table 1. Crude protein, crude fiber, neutral detergent fiber (NDF), acid detergent fiber (ADF), acid detergent lignin (ADL), calcium, total phosphorus, and amino acids were analyzed. Digestible energy (DE) was calculated based on the rabbit digestible energy data of individual ingredients provided in Nutrient Requirements of Meat Rabbits.

**Table 1:** Composition and nutrient levels of basal diet (air-dry basis).

Items	Content, %	Nutrients	Value, %
Corn	20.00	Digestible energy, (MJ/kg)^b^	10.75
Soybean meal(CP 43%)	15.00	Crude protein	16.31
Wheat bran	29.00	Crude fiber	16.24
Alfalfa meal (CP 17%)	20.00	Neutral detergent fiber	33.24
Rice husk powder	12.00	Acid detergent fiber	20.62
Limestone	1.50	Acid detergent lignin	5.12
CaHPO_4_	0.50	Calcium	1.11
NaCl	0.50	ATTD phosphorus	0.56
Premix^a^	1.50	Lysine	0.87
Total	100.00	Methionine+cysteine	0.62

^a^The Premix provided the following per kilogram of diet: VA 4000 IU, VD3 1000, VE 50 mg, choline 1 g.Fe (as ferrous sulfate 80 mg, Cu (as copper sulphate) 25mg, Zn(as zinc sulfate)70 mg, Mn 30 mg, Mg 200 mg, Robenidine hydrochloride,150 mg, Diclazuril)1 mg.

^b^DE was a calculated value, while the others were measured values.

### 2.5 Animal management

The experimental rabbits were housed in the same enclosed rabbit facility. 1 week before the trial, the housing and equipment were disinfected by flame sterilization and thoroughly cleaned. All rabbits were vaccinated against rabbit viral hemorrhagic disease and Pasteurella multocida with a combined vaccine administered subcutaneously at a dose of 1 mL per rabbit, following the standard immunization protocol. After ear tagging, the rabbits were housed individually by replicate in cages (50 cm × 50 cm × 50 cm), with four rabbits per cage. They were fed twice daily (08:00 and 18:00) under *ad libitum* feeding and drinking conditions. Supplementary lighting was provided using fluorescent lamps from 08:00 to 20:00 daily. The indoor temperature was maintained at 27 ± 2.18 °C, with a relative humidity of 67.50 ± 8.11%. The pens were cleaned daily to ensure a ventilated, sanitary, and dry environment.

### 2.6 Sample collection

At the end of the experiment, 30 experimental rabbits were selected for sample collection, with one rabbit randomly chosen from each of the six replicates per group. Blood samples (10 mL) were collected via cardiac puncture following a 12-h fast. After allowing the blood to stand for 20 min, the samples were centrifuged at 3,500 rpm for 15 min at 4 °C. The supernatant serum was aliquoted into 1.5 ml sterilized centrifuge tubes and stored at −20 °C for further analysis. Following blood collection, the rabbits were euthanized by air injection. The abdominal cavity was promptly opened, and the intestinal tract was separated. Cecal chyme was aseptically collected into 5 ml sterile cryovials and stored at −80 °C for subsequent analysis of cecal microbial diversity.

### 2.7 Determination indicators and methods

#### 2.7.1 Assay for serum inflammatory parameters

The concentrations of D-lactic acid (D-LA), diamine oxidase (DAO), interleukin-1β (IL-1β), and tumor necrosis factor-alpha (TNF-α) in serum were determined using enzyme-linked immunosorbent assay (ELISA) kits purchased from Nanjing Jiancheng Bioengineering Institute Co., Ltd. All procedures were performed in accordance with the manufacturer's instructions.

#### 2.7.2 Cecal microbial diversity analysis

##### 2.7.2.1 DNA extraction and PCR amplification

The extraction and sequencing of DNA from cecal chyme samples were completed by Beijing Allwegene Tech, Ltd (Beijing, China). Bacterial DNA was extracted using a PowerSoil DNA Isolation Kit (MoBio Laboratories, Carlsbad, CA, USA), following the manufacturer's instructions. The purity and quality of the genomic DNA were checked using 1% agarose gels. Bacterial DNA was PCR amplified with barcoded universal bacterial primers targeting the V3 and V4 variable regions of the 16S rRNA gene. After constructing the sequencing library, sequencing was performed on the Illumina NovaSeq platform.

##### 2.7.2.2 The bioinformatics analysis of the cecal microbiota

After sequencing, the raw data were stored in Fastq format. Quality control of the obtained Fastq data was performed using Pear (v0.9.11), Vsearch (v2.13.3), and QC-fasta-v2 (v2.0), ultimately yielding high-quality Fasta data. The high-quality Fastq data were then subjected to barcode and primer removal, followed by quality control and paired-end merging to obtain raw tags. The raw tags were further processed by removing chimeras and short sequences to obtain clean tags (high-quality sequences). Clustering analysis of clean-tags was performed using the Uparse clustering algorithm with QIIME (v1.8.0) and Vsearch (v2.13.3) software at 97% sequence identity to obtain operational taxonomic units (OTUs), which were then annotated. Alpha diversity analysis, beta diversity analysis, and LEfSe (Linear discriminant analysis Effect Size) were used to analyze sample composition and differences in community structure among samples.

### 2.8 Statistical analysis

Data were analyzed using one-way ANOVA (SPSS 22.0, IBM SPSS, Chicago), and the results were expressed as the mean ± standard deviation. Duncan's method was used for multiple comparisons between each group. The *P* < 0.05 was considered statistically significant, and the *P* < 0.01 was considered highly significant.

## 3 Results

### 3.1 Effects of 1,8-Cineole and α-Terpineol from *Cinnamomum longepaniculatum* on serum inflammatory cytokine factors of meat rabbits

Table 2 indicates that the D-LA concentration was markedly reduced in the ANT, TEP2, and CNI groups relative to the control group (*P* < 0.01), and also significantly lower in the CNI+TEP2 group (*P* < 0.05). No significant difference in D-LA content was observed among the ANT, TEP2, and CNI groups (*P* > 0.05); however, the D-LA content in the CNI + TEP2 group was considerably elevated compared to the ANT, TEP2, and CNI groups (*P* < 0.05). In comparison to the CON group, the DAO content was markedly reduced in the ANT, TEP2, and CNI groups (*P* < 0.01), as well as in the TEP2 + CNI group (*P* < 0.05). The DAO content in the TEP2, CNI, and CNI + TEP2 groups was significantly higher than in the ANT group *(P* < 0.01). The IL-1β concentrations in the ANT, TEP2, and CNI groups were markedly reduced compared to the CON group (*P* < 0.01). The IL-1β levels in the CNI + TEP2 group were lower than those in the CON group; however, the difference was not statistically significant (*P* > 0.05). Compared with the CON group, TNF-α levels were markedly reduced in the ANT and CNI groups *(P* < 0.01) and in the TEP2 group (*P* < 0.05). The CNI+TEP group showed a declining trend compared with the CON group; however, the difference was not statistically significant (*P* > 0.05). The D-LA levels in the TEP2, CNI, and TEP2+CNI groups, as well as the IL-1β level in the CNI group, showed no statistically significant differences compared to the ANT group (*P* > 0.05).

**Table 2:** Effects of 1,8-cineole and α-terpineol from *Cinnamomum longepaniculatum* on serum inflammatory cytokine factor of meat rabbits.

Groups	D-LA (μmol/ml)	DAO (U/L)	IL-1β (ng/L)	TNF-α (ng/L)
CON	0.12 ± 0.03^Aa^	19.00 ± 1.21^Aa^	22.98 ± 1.57^Aa^	125.78 ± 15.94^Aa^
ANT	0.07 ± 0.02^Bbc^	8.10 ± 0.64^Cd^	15.48 ± 1.08^Bc^	84.66 ± 10.74^Cc^
TEP2	0.06 ± 0.01^Bc^	11.95 ± 0.74^Bc^	17.56 ± 1.41^Bb^	105.92 ± 13.26^ABb^
CNI	0.08 ± 0.03^Bbc^	11.31 ± 4.63^BCc^	16.69 ± 2.19^Bbc^	104.02 ± 14.63^BCb^
TEP2+CNI	0.09 ± 0.02^ABb^	16.14 ± 2.5^Ab^	22.01 ± 1.29^Aa^	115.01 ± 13.29^ABab^
*P*	< 0.001	< 0.001	< 0.001	< 0.001

In the same column, values with no letter or the same letter superscripts mean no significant difference (*p*> 0.05), while values with different small letter superscripts mean substantial difference (*p* < 0.05), and with different capital letter superscripts mean significant difference (*p* < 0.01)—the same as Table 3.

### 3.2 Effects of α-terpineol and 1,8-cineole from *Cinnamomum longepaniculatum* on cecal microbial diversity of meat rabbits

#### 3.2.1 Venn diagram based on OTU

**Figure 1:**
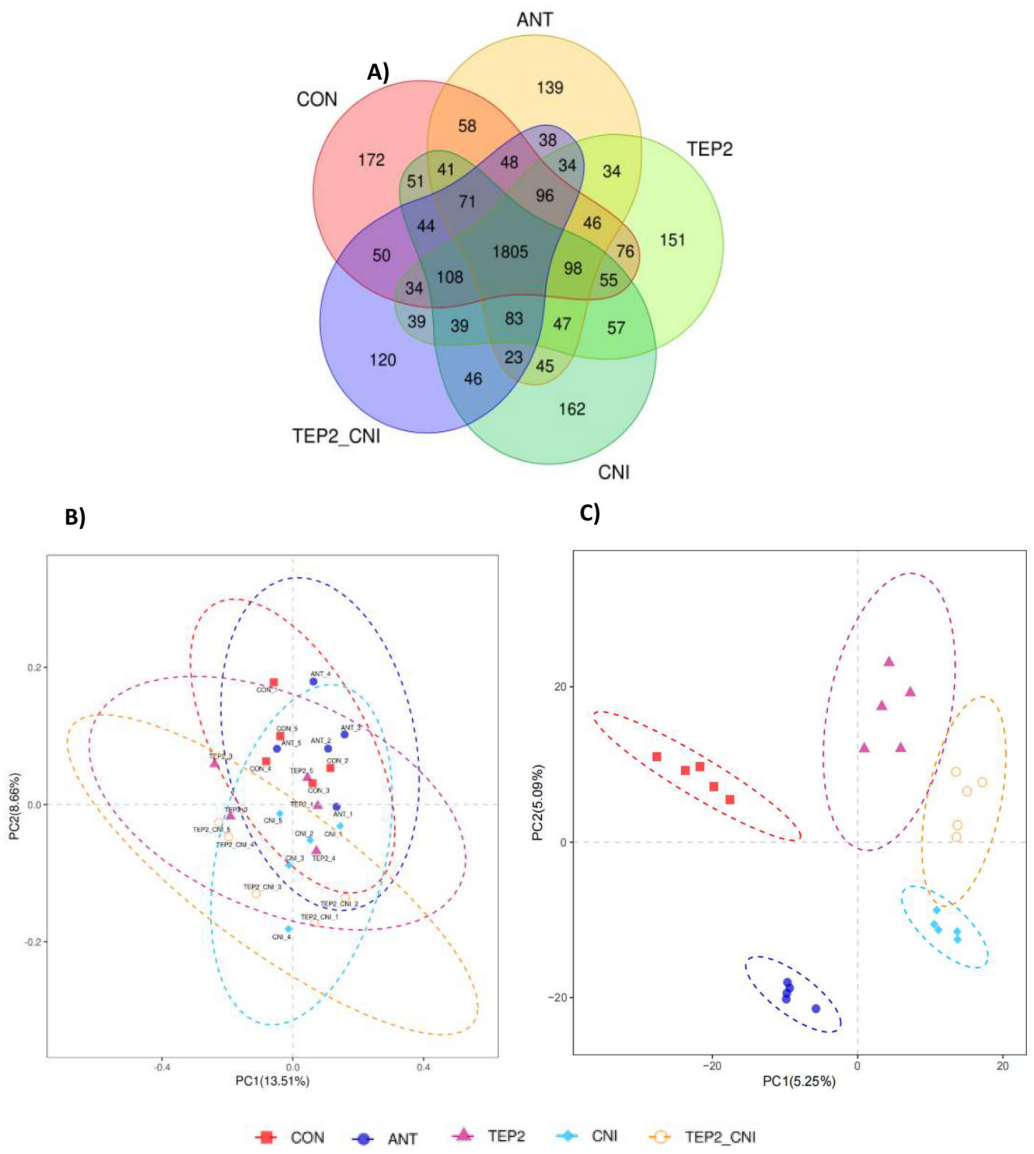
OTU sharing and community structure. **(A)** Venn diagram illustrating the shared and distinct OTUs among five groups: CON, ANT, TEP2, CNI, and TEP2+CNI. All groups shared the basic OTUs, although each group possessed distinct OTUs. **(B)** Principal Coordinates Analysis (PCoA) based on both UniFrac and Bray-Curtis distance matrices of OTUs. The plot illustrates variations in microbial community composition among the five treatment groups. The first two principal components (PC1 = 13.51%, PC2 = 8.66%) together explained 22.17% of the total variance. **(C)** Partial Least Squares Discrimination Analysis (PLS-DA) based on OTUs.PLS-DA was used to model the correlation between microbial abundance and sample categories for group prediction and classification. The initial two components represented 20.34% of the variance.

Figure 1A illustrates a Venn diagram that shows the similarities and differences between the operational taxonomic units (OTUs) from the five sets of experiments: CON, ANT, TEP2, CNI, and TEP2 + CNI. Consistent with a highly homogeneous microbial community structure, all groupings shared 1,805 core OTUs. The presence of unique OTUs in each treatment group indicated microbial diversity in response to those treatments. In CON, there were 172 distinct OTUs; in ANT, 139; in TEP2, 151; in CNI, 162; and in TEP2 + CNI, 120. With fewer distinct OTUs, the TEP2 + CNI group likely had more microbial overlap than the other groups. When compared, the CON and CNI groups had significantly more unique OTUs, suggesting that their microbial profiles were more distinct. The results show that the microbial communities in the treatment groups are similar and distinct, with various chemicals having diverse effects on microbial diversity.

#### 3.2.2 Alpha diversity analysis

Table 3 shows that species richness decreased substantially following antibiotic treatment, as the observed species index in the ANT group was lower than that in the CON group (*P* < 0.05). The observed species index likewise trended lower in the TEP2, CNI, and TEP2 + CNI groups compared with the CON group, although these differences were not statistically significant (*P* > 0.05). Compared to the CON, ANT, and TEP2 + CNI groups, the TEP2 group had a substantially higher Chao1 index (*P* < 0.05), indicating that the estimated species richness was enhanced by TEP2 supplementation. After receiving CNI therapy, microbial diversity improved, as evidenced by a substantially higher Shannon index in the CNI group compared with the ANT group (*P* < 0.05). Among the other groups, there were no notable variations in the Shannon or Simpson indices (*P* > 0.05). Taken together, our findings provide evidence that TEP2 and CNI therapies partially reversed the effects of antibiotic exposure on microbial diversity and richness.

**Table 3:** Alpha diversity indexes of cecal microbial flora.

Groups	Observed_species index	Chao1 index	Shannon index	Simpson index
CON	1733.58 ± 64.85^a^	2082.97 ± 73.36^ab^	7.86 ± 0.22^a^	0.98 ± 0.02
ANT	1633.16 ± 79.26^b^	2009.71 ± 92.95^b^	7.48 ± 0.28^b^	0.97 ± 0.01
TEP2	1724.00 ± 30.52^ab^	2133.38 ± 52.39^a^	7.81 ± 0.19^a^	0.98 ± 0.01
CNI	1657.54 ± 63.15^ab^	2062.88 ± 108.41^ab^	7.9 ± 0.31^a^	0.99 ± 0.01
TEP2+CNI	1644.2 ± 75.65^ab^	2004.93 ± 66.2^6b^	7.73 ± 0.15^ab^	0.98 ± 0.01
*P*	0.041	0.039	0.044	0.173

#### 3.2.3 Beta diversity analysis

To evaluate the beta diversity of microbial communities across different treatment groups, both unsupervised principal coordinates analysis (PCoA) and supervised PLS-DA were applied. PCoA was performed based on both UniFrac and Bray-Curtis distance matrices to characterize overall variations in microbial community composition.

Figure 1B shows that the first two principal components (PC1 and PC2) explained 13.51 and 8.66% of the total variance, respectively, with a combined contribution of 22.17%. The ordination plot showed partial separation of samples according to treatment, indicating that the different treatments altered the overall structure of the gut microbial communities.

Figure 1C presents the grouping differentiation of microbial communities by supervised Partial Least Squares Discriminant Analysis (PLS-DA). Along the first two principal components (PC1 = 15.25%, PC2 = 5.09%), the ANT, TEP2, CNI, and TEP2 + CNI groups were clearly separated from each other, indicating distinct microbial community composition profiles under different treatments. Replicate samples within the ANT and CNI groups clustered closely together, reflecting good reproducibility and low intra-group variation. In comparison, the CON, TEP2, and TEP2 + CNI groups showed relatively scattered sample distribution, suggesting higher heterogeneity in community structure within these groups.

#### 3.2.4 Effects of 1,8-cineole and α-terpineol from *Cinnamomum longepaniculatum* on the relative abundance of cecal microbiota in meat rabbits

##### 3.2.4.1 Distribution of cecal microbial flora of meat rabbits at the phylum level

**Figure 2:**
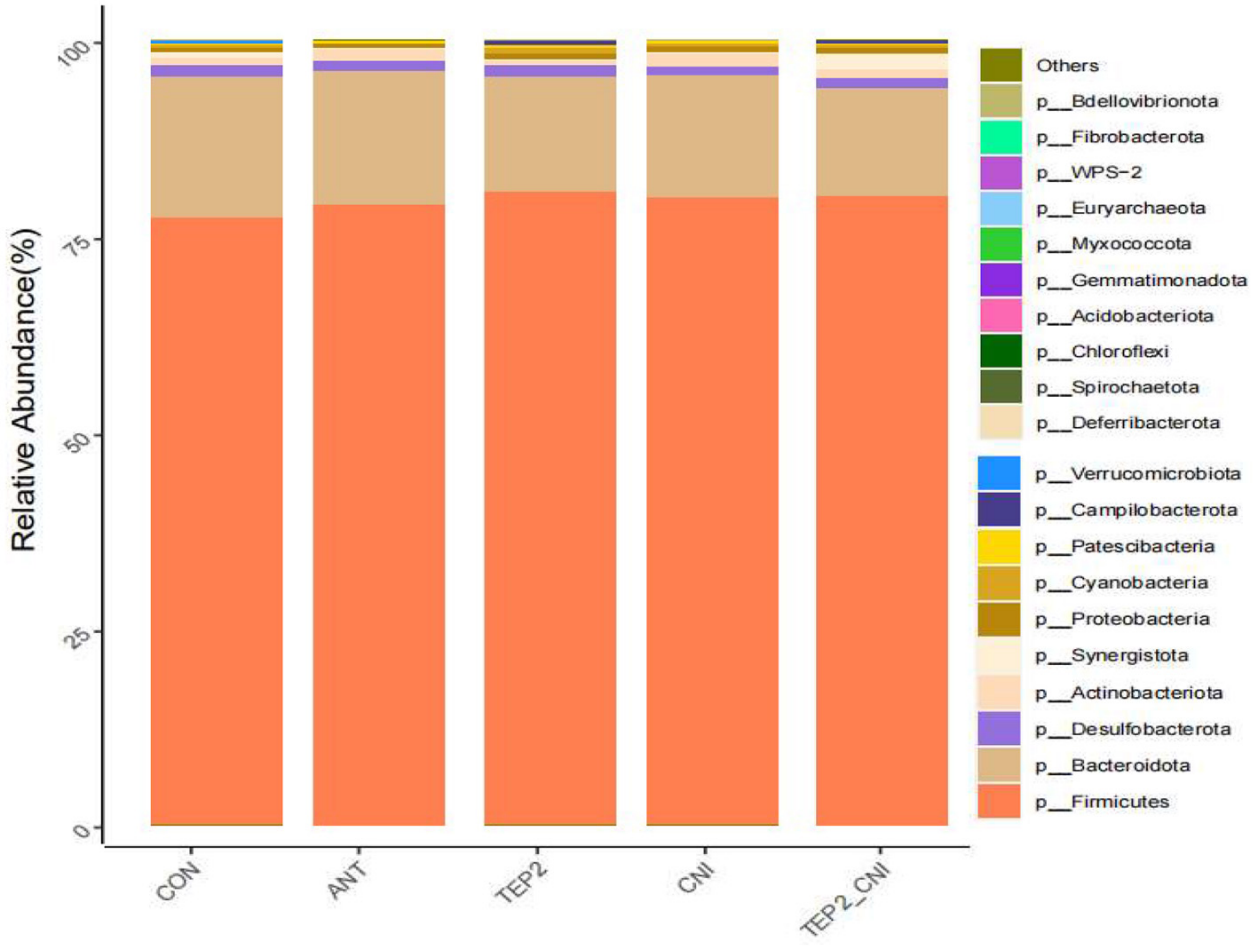
Cecal microbiota at the phylum level. Bar graph illustrating the relative abundance of predominant bacterial phyla in the cecum of rabbits subjected to various treatments. Firmicutes and Bacteroidota predominated across all categories, with variations noted in other taxa under distinct treatments.

Firmicutes and Bacteroidetes phyla accounted for over 93% of the overall bacterial abundance in the cecal microbial community of meat rabbits across all treatment groups (Table 4 and Figure 2). These two predominant phyla accounted for 95.29% in the CON group, 96.02% in the ANT group, 95.36% in the TEP2 and CNI group, and 93.49% in the TEP2 + CNI group. Compared with the control group, the antibiotic group showed a notable decline in Verrucomicrobiota (*P* < 0.05) and a notable increase in Actinobacteriota (*P* < 0.05). The TEP2 group exhibited a noteworthy rise in Cyanobacteria and Campilobacterota (*P* < 0.01), whereas the CNI group showed higher levels of Actinobacteriota (*P* < 0.01). There was a significant increase in Actinobacteriota and Synergistota in the TEP2 + CNI group (*P* < 0.05), as well as a considerable rise in Campilobacteria (*P* < 0.01). In TEP2, the abundance of Actinobacteriota was much lower than in the ANT group, but in TEP2 + CNI, the abundance of Synergistota was significantly higher (*P* < 0.01). Proteobacteria abundance was greater in the CNI group compared to the ANT group (*P* < 0.05), but no significant differences were seen in the other groups (*P* > 0.05). Furthermore, compared to all other treatments, the CON group had a considerably higher abundance of Deferribacterota (*P* < 0.01). The results show that the phylum-level composition of the cecal microbiota was altered by chemical supplementation and antibiotic exposure. Some bacterial groups were restored or modulated by TEP2 and CNI treatments. In the same row, values with no letter or the same letter superscripts mean no significant difference (*P* > 0.05), while values with different small letter superscripts mean considerable difference (*P* < 0.05), and values with different capital letter superscripts mean significant difference (*P* < 0.01)—the same as Table 4.

**Table 4:** Distribution of cecal microbial flora of meat rabbits at the phylum level.

Items	Groups					*P-value*
	CON	ANT	TEP2	CNI	TEP2+CNI	
Firmicutes	77.44 ± 3.43	79.06 ± 5.12	80.6 ± 5.29	79.91 ± 3.08	80.11 ± 4.41	0.802
Bacteroidota	17.85 ± 3.60	16.96 ± 5.06	14.76 ± 5.36	15.54 ± 3.31	13.8 ± 4.45	0.613
Desulfobacterota	1.53 ± 0.37	1.36 ± 0.42	1.32 ± 0.41	1.21 ± 0.4	1.31 ± 0.48	0.713
Actinobacteriota	0.83 ± 0.52^c^	1.48 ± 0.49^a^	0.73 ± 0.38^c^	1.56 ± 0.41^a^	1.01 ± 0.35^ab^	0.019
Synergistota	0.73 ± 0.66^ABb^	0.17 ± 0.1^Bb^	0.23 ± 0.23^Bb^	0.21 ± 0.05^Bb^	2.05 ± 1.66^Aa^	0.006
Proteobacteria	0.63 ± 0.28^ab^	0.46 ± 0.14^b^	0.56 ± 0.19^ab^	0.82 ± 0.12^a^	0.67 ± 0.34^ab^	0.191
Cyanobacteria	0.24 ± 0.2^Bb^	0.15 ± 0.04^Bb^	0.82 ± 0.27^Aa^	0.26 ± 0.12^Bb^	0.42 ± 0.37^Bb^	< 0.001
Patescibacteria	0.32 ± 0.09	0.25 ± 0.07	0.31 ± 0.22	0.35 ± 0.06	0.21 ± 0.08	0.352
Campilobacterota	0.03 ± 0.03^Cc^	0.06 ± 0.03^Cc^	0.49 ± 0.19^Aa^	0.05 ± 0.03^Cc^	0.27 ± 0.13^Bb^	< 0.001
Verrucomicrobiota	0.25 ± 0.15^a^	0.03 ± 0.02^b^	0.16 ± 0.11^ab^	0.06 ± 0.04^b^	0.13 ± 0.12^ab^	0.021
Deferribacterota	0.11 ± 0.04^Aa^	0 ± 0^Bb^	0 ± 0.01^Bb^	0 ± 0^Bb^	0 ± 0^Bb^	< 0.001

In the same column, values with no letter or the same letter superscripts mean no significant difference (*p*> 0.05), while values with different small letter superscripts mean substantial difference (*p* < 0.05), and with different capital letter superscripts mean significant difference (*p* < 0.01)—the same as Table 3.

##### 3.2.4.2 Distribution of cecal microbial flora of meat rabbits at the genus level

**Figure 3:**
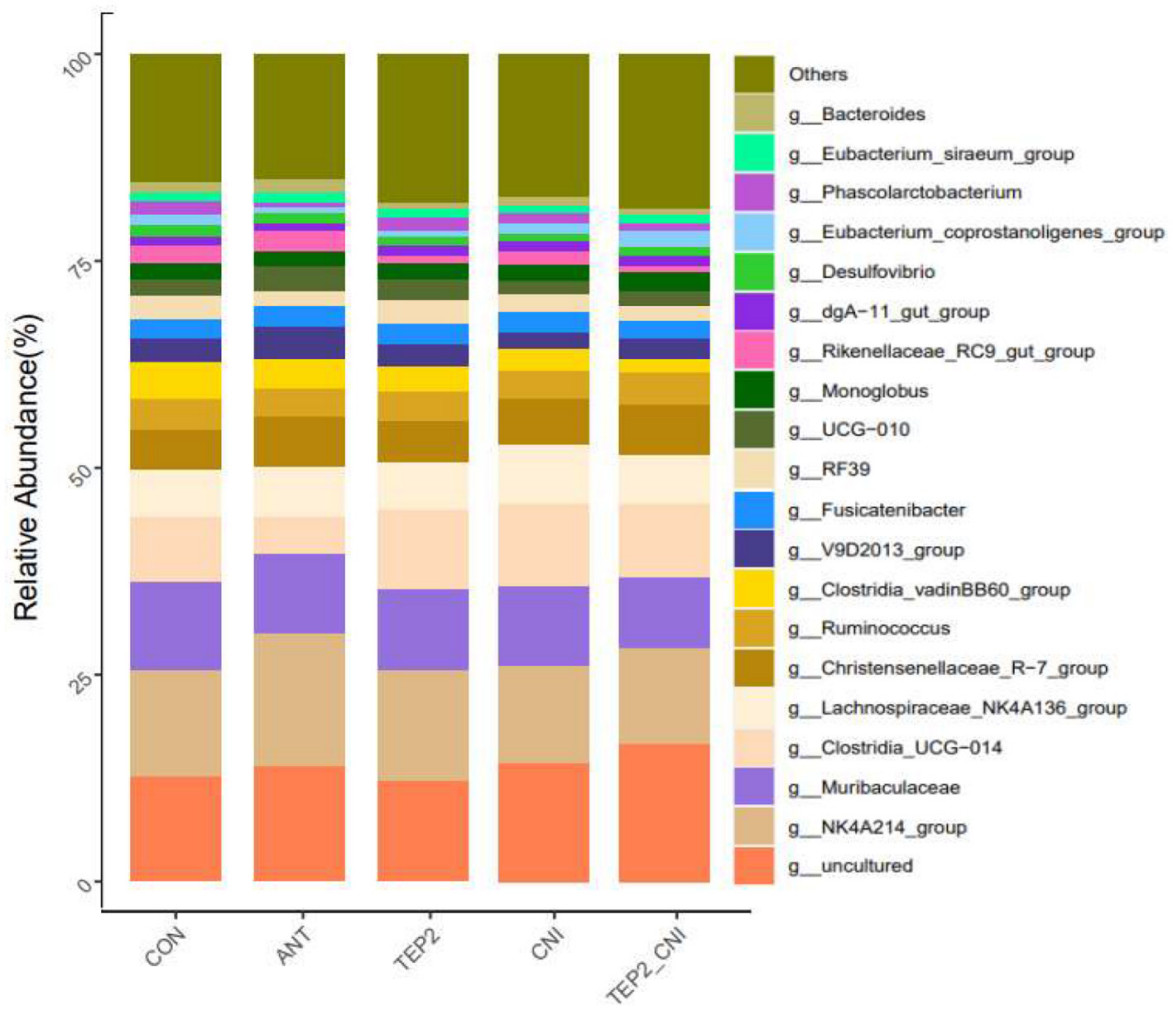
Cecal microbiota at the genus level. Bar graph illustrating the relative abundance of predominant bacterial species in the cecum of rabbits. Prominent genera encompass Unclassified taxa, NK4A214 group, and Muribaculaceae, exhibiting treatment-specific alterations in several genera.

In the cecal microbiota of meat rabbits, the most abundant genera were Ruminococcus, Unclassified, Muribaculaceae, Clostridia UCG-014, Lachnospiraceae NK4A136, Christensenellaceae R-7, and NK4A214, as demonstrated in Table 5 and Figure 3. No other group accounted for more than 3% of the total bacterial abundance. The different groups did not show a substantially greater relative abundance of Unclassified bacteria than the TEP2 + CNI group (*P* < 0.05), indicating that the combination treatment enriched uncharacterized taxa. The antibiotic-induced suppression of the Clostridia UCG-014 genus was demonstrated by significantly lower abundance in the ANT group compared to the other groups (*P* < 0.05). Compared with the CNI and TEP2 + CNI groups, the CON and ANT groups showed a considerably higher abundance of the Clostridia vadinBB60 group (*P* < 0.01). In comparison to the TEP2, CNI, and TEP2 + CNI groups, the ANT group exhibited a considerably higher abundance of the Rikenellaceae RC9 gut group (*P* < 0.05). In comparison to the ANT and TEP2 groups (*P* < 0.01), the TEP2 + CNI group had a considerably greater concentration of the Eubacterium coprostanoligenes group, and it was also higher than in the CON and CNI groups (*P* < 0.05). However, when comparing the ANT group to the CON and TEP2 groups, the Phascolarctobacterium genus was found to be much less abundant (*P* < 0.05). When comparing the ANT group with CNI and TEP2 + CNI, no significant differences were observed (*P* > 0.05). All things considered, our findings indicate that antibiotic therapy altered the abundance of a few key genera; however, supplementation with TEP2 and CNI, particularly when combined, helped restore several beneficial taxa and diversified the gut microbiome.

**Table 5:** Distribution of cecal microbial flora of meat rabbits at the genus level.

Items	Groups				*P*值	
	CON	ANT	TEP2	CNI	TEP2 + CNI	*P-value*
Unclassified	12.76 ± 3.54^b^	14.00 ± 1.45^ab^	12.21 ± 2.98^b^	14.31 ± 1.88^ab^	16.76 ± 3.12^a^	0.117
NK4A214 group	12.83 ± 3.26	16.00 ± 2.29	13.34 ± 4.69	11.8 ± 3.42	11.44 ± 2.86	0.268
*Muribaculaceae*	10.76 ± 3.64	9.59 ± 4.90	9.87 ± 4.23	9.52 ± 2.97	8.64 ± 5.50	0.959
Clostridia UCG 014	7.75 ± 2.52^a^	4.51 ± 1.02^b^	9.64 ± 2.72^a^	10.13 ± 2.77^a^	8.92 ± 2.56^a^	0.04
Lachnospiraceae NK4A136 group	5.78 ± 2.28	6.01 ± 1.36	5.62 ± 1.2	7.11 ± 2.42	5.96 ± 3.92	0.881
Christensenellaceae R-7 group	4.75 ± 1.32	6.11 ± 3.25	4.93 ± 0.76	5.59 ± 1.40	5.92 ± 3.59	0.859
*Ruminococcus*	3.69 ± 1.04	3.38 ± 1.44	3.73 ± 2.50	3.42 ± 1.31	3.96 ± 2.39	0.986
Clostridia vadinBB60 group	4.52 ± 0.64^Aa^	3.68 ± 0.87A^Bab^	2.9 ± 1.18^BCbc^	2.5 ± 0.87^BCcd^	1.64 ± 0.42^Cd^	< 0.001
V9D2013 group	2.91 ± 1.11	3.79 ± 2.84	2.82 ± 2.08	2.03 ± 1.41	2.39 ± 1.54	0.652
*Fusicatenibacter*	2.18 ± 1	2.48 ± 1.08	2.39 ± 0.56	2.41 ± 1.09	2.22 ± 0.82	0.982
RF39	2.88 ± 0.68	1.93 ± 1.18	2.81 ± 0.59	2.2 ± 0.68	1.83 ± 0.37	0.113
UCG-010	2.1 ± 0.69	2.87 ± 1.04	2.46 ± 1.21	1.6 ± 0.85	1.64 ± 0.6	0.201
*Monoglobus*	1.79 ± 0.45	1.84 ± 0.71	2.1 ± 0.55	1.91 ± 0.32	2.42 ± 0.65	0.396
Rikenellaceae RC9 gut group	2.1 ± 1.14^ab^	2.41 ± 1.06^a^	0.86 ± 0.54^bc^	1.65 ± 1.18^abc^	0.66 ± 0.36^c^	0.028
dgA-11 gut group	1.22 ± 0.39	0.97 ± 0.9	1.17 ± 0.59	1.19 ± 0.76	1.31 ± 0.82	0.960
*Desulfovibrio*	1.32 ± 0.35	1.25 ± 0.39	1.13 ± 0.47	1.03 ± 0.39	1.08 ± 0.48	0.498
Eubacterium coprostanoligenes group	1.3 ± 0.46^ABb^	0.67 ± 0.17^Bc^	0.75 ± 0.22^Bbc^	1.17 ± 0.45^ABbc^	1.88 ± 0.58^Aa^	< 0.001
*Phascolarctobacterium*	1.57 ± 0.68^a^	0.47 ± 0.29^b^	1.5 ± 0.51^a^	1.22 ± 0.89^ab^	0.97 ± 0.69^ab^	0.044
Eubacterium_siraeum_ group	1.11 ± 0.39	1.38 ± 0.9	1.15 ± 0.85	0.81 ± 0.24	1.02 ± 0.36	0.686
*Bacteroides*	1.26 ± 0.39	1.52 ± 0.97	0.67 ± 0.29	1.25 ± 0.75	0.66 ± 0.31	0.127

In the same row, values with no letter or the same letter superscripts mean no significant difference (*p*> 0.05), while those with different small letter superscripts mean significant difference (*p* < 0.05), and with different capital letter superscripts mean significant difference (*p* < 0.01)—the same as Table 5.

**Figure 4:**
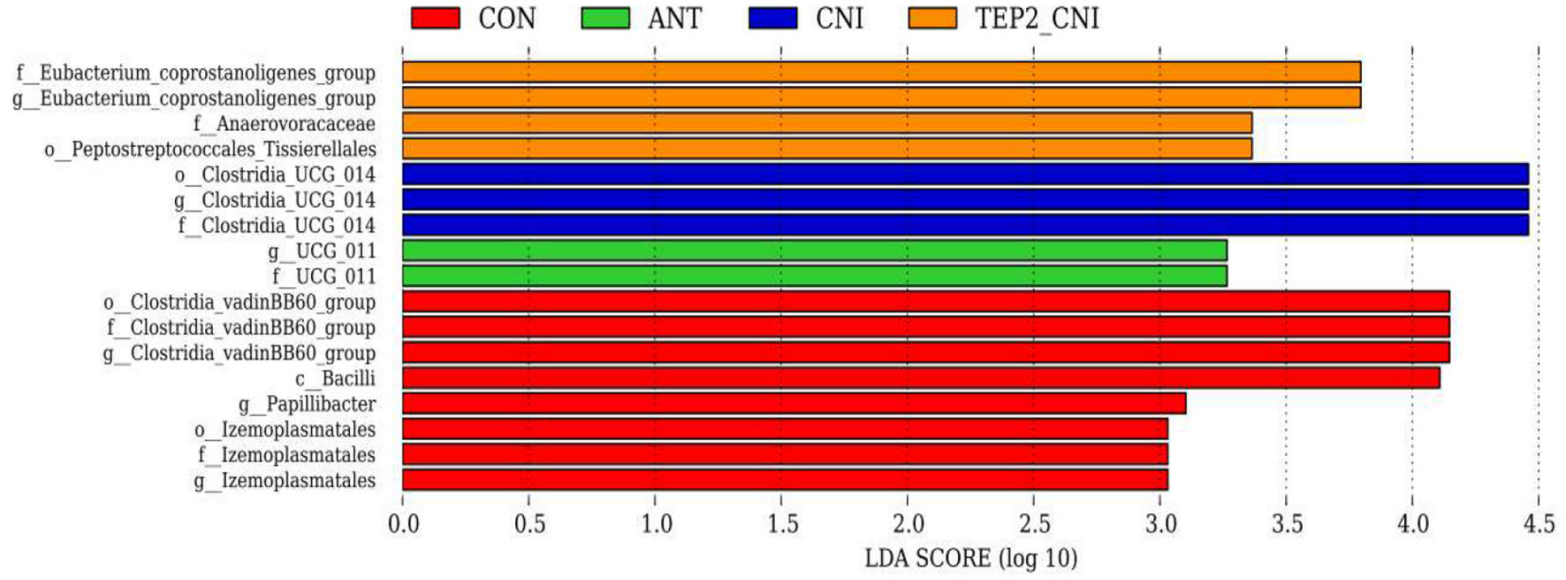
LEfSe analysis of differential taxa. LEfSe study demonstrating significant differences in bacterial taxa among groups. Numerous species were identified as exclusive to specific treatments, highlighting treatment-specific microbial patterns.

To further identify taxa with significant abundance differences between groups, a Linear Discriminant Analysis (LDA) Effect Size (LEfSe) was performed on the gut microbiota. Figure 4 displays the major contributing taxa that show significant differences in microbial diversity among the groups. Specifically, eight taxa were identified in the CON group, two in the ANT group, three in the CNI group, and four in the TEP2 + CNI group, while none were identified in the TEP2 group.

## 4 Discussion

The worldwide transition from antibiotic growth promoters in livestock nutrition has necessitated the development of scientifically substantiated, multifaceted alternatives that enhance both animal health and productivity (27). The EOs generated from plants and their bioactive components have emerged as attractive candidates due to their antibacterial, anti-inflammatory, and immunomodulatory properties (28). The intrinsic complexity of complete EOs often conceals the unique mechanisms and roles of individual constituents (29). This study extracted and examined the effects of two primary monoterpenoids from *C. longepaniculatum* essential oil-−1,8-cineole and α-terpineol—on important indices of intestinal health in meat rabbits. Utilizing a comparative approach encompassing individual, combination, and antibiotic-based therapies, our results provide a definitive mechanistic understanding of how these purified chemicals influence systemic inflammation and cecal microbial ecology. The findings provide a sophisticated framework for developing advanced phytogenic feed additives that aim to enhance gut barrier integrity, immunological regulation, and microbial balance.

### 4.1 Effects of 1,8-cineole and α-terpineol on serum inflammatory cytokine levels in meat rabbits

The integrity of the gastrointestinal barrier is a crucial factor in systemic health, serving as the primary defense against the entry of luminal antigens, endotoxins, and microbial metabolites into the bloodstream (30). The breach of this barrier triggers a series of metabolic and immunological disruptions that adversely affect growth performance and overall wellbeing (31). The current investigation demonstrates a notable decrease in blood levels of D-lactic acid (D-LA) and diamine oxidase (DAO) in rabbits receiving supplementation with 1,8-cineole, α-terpineol, or antibiotics, thereby providing direct evidence of improved intestinal barrier integrity. D-LA, a metabolic byproduct of bacterial fermentation, and DAO, an intracellular enzyme prevalent in mature enterocytes, are recognized as biomarkers of intestinal permeability (32). Their heightened plasma levels are directly associated with augmented intestinal permeability and mucosal injury. The significant reduction in these indicators across all active treatment groups, especially with the supplementation of individual 1,8-cineole or α-terpineol, robustly indicates a stabilizing effect on the intestinal epithelium. This discovery aligns with *in vitro* studies indicating that tea tree oil, rich in these monoterpenoids, can alleviate lipopolysaccharide (LPS)-induced barrier impairment in intestinal epithelial cells by enhancing tight junction proteins (33). Our *in vivo* findings validate this model, demonstrating that 1,8-cineole and α-terpineol confer cellular protective effects, resulting in quantifiable enhancements in gut barrier integrity within a production animal model. Concomitant with the enhancement of barrier indicators, we observed a substantial downregulation of key proinflammatory cytokines. Serum concentrations of interleukin-1β (IL-1β) and tumor necrosis factor-alpha (TNF-α) were significantly diminished in rabbits administered 1,8-cineole or α-terpineol alone, exhibiting efficacy comparable to that of the antibiotic control. This anti-inflammatory effect is of critical physiological significance. TNF-α and IL-1β serve as principal regulators of the inflammatory cascade; their overexpression results not only from barrier dysfunction but also acts as a direct catalyst for additional mucosal damage, epithelial cell death, and metabolic problems associated with chronic inflammation (34, 35). The capacity of these plant-derived monoterpenoids to inhibit these cytokines to a degree akin to that of strong antibiotics highlights their considerable immunomodulatory potential. The mechanism likely involves the suppression of core proinflammatory signaling pathways. 1,8-Cineole is a recognized inhibitor of the nuclear factor-kappa B (NF-κB) and mitogen-activated protein kinase (MAPK) pathways, which are essential for the transcriptional activation of TNF-α, IL-1β, and IL-6 (36, 37). α-Terpineol, recognized for its antibacterial properties, also exhibits significant anti-inflammatory properties, likely mediated by modulation of the Nrf2 antioxidant pathway and the accompanying decrease in oxidative stress (38).

The combined use of drugs can exhibit effects such as synergism, additive effect, and antagonism. This study reveals a significant and unexpected finding regarding the varying effectiveness of solo vs. combination supplementation. The individual drugs at 80 mg/kg exhibited substantial anti-inflammatory and barrier-strengthening effects; however, the combination treatment (40 mg/kg each) resulted in a diminished response. The combined use of α-Terpineol and 1,8-Cineole exhibited an antagonistic effect, which may be attributed to their different properties and pharmacological actions. TEP is a monocyclic monoterpenoid tertiary alcohol with a lilac aroma (39), whereas CNI is a bicyclic monoterpenoid compound with a slight camphor-like odor and a pungent, cool taste (40). Studies have shown that CNI and TEP differ considerably in their antibacterial and antioxidant functions. TEP exhibits higher scavenging rates against 1,1-diphenyl-2-picrylhydrazyl (DPPH·) and 22′-azino-bis (3-ethylbenzothiazoline-6-sulfonic acid) diammonium salt (ABTS+·) free radicals compared to CNI, and the inhibition zones of TEP against Escherichia coli, Salmonella, and Staphylococcus aureus are larger than those of CNI (33). CNI has a more pronounced anti-inflammatory effect and is an important drug for the treatment of respiratory and cardiovascular diseases (41). CNI is lipophilic, can interfere with intracellular lipid synthesis, and increase drug permeability. Its anti-inflammatory mechanism is reported to be mainly associated with the inhibition of the nuclear factor-κB (NF-κB) and nuclear factor erythroid 2-related factor 2 (Nrf2) signaling pathways (40, 41). Both monoterpenes competitively inhibit CYP2B1 (IC50: 4.7 μm for 1,8-cineole and 14.8 μm for terpineol) and can inhibit P-glycoprotein, potentially reducing absorption or altering metabolism of each other. TEP and CNI can regulate the NF-κB and Nrf2 pathways. A threshold effect cannot be excluded: each compound required 80 mg/kg to be effective, but the combination gave only 40 mg/kg each. If each needs >50 mg/kg to trigger sustained anti-inflammatory signaling, the combined regimen would produce apparent antagonism without true biological interaction. Future studies should measure plasma drug levels and perform factorial dose-response analyses to distinguish among these possibilities. This finding suggests a non-linear or possibly competitive interaction when both drugs are administered simultaneously at these specific levels. Potential explanations encompass pharmacokinetic interactions influencing absorption or metabolism, competition for analogous biological targets or signaling pathways, or a threshold effect necessitating a minimum effective dose of each agent for maximum efficacy. This highlights a fundamental aspect of phytogenic formulations: the combination of bioactive substances does not automatically confer additive or synergistic benefits. This may result in antagonism, underscoring the need for accurate, evidence-based dosing techniques (38).

The discrepancy between our findings and other research indicating increased cytokine levels after essential oil intake highlights the context-dependent characteristics of phytogenic effects (42). The discrepancies can be ascribed to various factors: the use of complex, whole EOs rather than purified compounds; notable variations in dosage; the health status and species of the experimental animals; and the duration of supplementation. Our research, which utilizes pure chemicals in a healthy rabbit model at a specified dosage, elucidates their inherent anti-inflammatory properties, thereby eliminating the confounding effects of other oil components.

### 4.2 Effects of α-terpineol and 1,8-cineole on cecal microbial diversity in meat rabbits

The mammalian gut microbiota is a complex ecosystem that is essential for host digestion, metabolism, immunological development, and resistance to pathogens (43). In herbivores such as rabbits, the cecal microbiota plays a crucial role in digesting fibrous substances and assimilating nutrients (44). Our sequencing data corroborate that the cecal microbiome of meat rabbits is primarily constituted of the phyla Firmicutes and Bacteroidota, aligning with previous studies. The exceptional stability of these two predominant phyla, which together comprise over 93% of the total relative abundance across all groups, indicates the ecological robustness of the core microbial community in healthy adult animals (45, 46). Dietary treatments can influence the gut microbiota, typically not significantly altering the dominant phyla but rather affecting the relative abundances of subordinate taxa and community diversity (47). Our findings corroborate this notion, indicating that although the fundamental structure remained unaltered, 1,8-cineole and α-terpineol prompted substantial alterations in microbial diversity and composition at more granular taxonomic levels. Alpha diversity measurements, which indicate species richness and evenness within a sample, showed treatment-specific effects. The injection of antibiotics consistently diminished species richness (Observed species index), a typical result of broad-spectrum antimicrobial effects. Conversely, supplementation with α-terpineol (TEP2 group) resulted in a markedly elevated Chao1 index, an indicator of overall species richness, compared with the antibiotic and combination groups. This suggests that α-terpineol not only mitigated the diversity-reducing impact of antibiotics but also promoted an environment favorable to a more abundant microbial community. Likewise, the CNI showed a markedly elevated Shannon index compared with the antibiotic group, indicating greater overall microbial diversity and evenness. The potential of these phytogenics to augment or maintain microbial diversity presents a significant benefit over conventional antibiotics, as increased gut microbial diversity is commonly linked to enhanced ecosystem stability, functional redundancy, and host health resilience (46). PCoA results based on both UniFrac and Bray-Curtis distances from beta diversity analysis indicated that different treatments significantly altered the overall structure of gut microbial communities. Furthermore, PLS-DA revealed that several treatment groups exhibited relatively scattered sample distribution, suggesting higher heterogeneity in microbial community structure within these groups. It should be acknowledged that PLS-DA is a supervised ordination method that artificially maximizes inter-group separation and may be prone to overfitting under limited sample sizes. Therefore, the community structural differences observed in this study were comprehensively verified by combining unsupervised PCoA (UniFrac and Bray-Curtis distances) and supervised PLS-DA results, which further guaranteed the reliability of the grouping patterns. Overall, antibiotic exposure and chemical supplementation markedly reshaped the gut microbial community structure. In comparison, the ANT and CNI treatments maintained relatively stable and consistent microbial community composition patterns with low intra-group variation.

In addition to diversity indices, our analysis revealed significant compositional alterations at both the phylum and genus levels, which closely align with the reported increases in systemic inflammation. A noteworthy discovery was the substantial increase in the phylum Actinobacteriota across all treatment groups that received antibiotics, 1,8-cineole, and the composite essential oil. This phylum encompasses advantageous taxa, including Bifidobacterium, which are recognized for their contributions to gut homeostasis, the production of short-chain fatty acids, and immunomodulation (18). The enhancement of Actinobacteriota by these therapies indicates a direct microbial mechanism that promotes better gut health. In this study, the relative abundance of Cyanobacteria in the TEP2 group was significantly increased, which could be excluded as being caused by exogenous contamination. First, from the dominant genera in the cecal contents of this study, all dominant cecal genera (including Unclassified NK4A214 group, Muribaculaceae, Clostridia UCG-014, Lachnospiraceae NK4A136 group, Ruminococcus, Fusicatenibacter, Bacteroides, and others) were typical indigenous anaerobic gut microbes; no photosynthetic environmental cyanobacteria (e.g., Microcystis) were detected. Second, the enriched Cyanobacteria were non-photosynthetic anaerobic Melainabacteria, and only the TEP2 group showed an elevation, which was inconsistent with the characteristics of contamination. The significant enrichment of Cyanobacteria in the TEP2 group might be related to the selective regulation of α-Terpineol. α-Terpineol targeted and inhibited Bacteroidota, Actinobacteriota, and Deferribacterota, thereby reducing nutrient competition and niche exclusion, and releasing ecological niches for Cyanobacteria.

Simultaneously, we noted a substantial reduction in the phylum Deferribacterota. Deferribacterota, although often few in number in healthy intestines, has been commonly linked to intestinal inflammation and dysbiosis across multiple disease models (48, 49). The decrease in this inflammation-associated phylum offers a credible microbiological association for the simultaneous decline in serum inflammatory markers. Additionally, a decrease in Proteobacteria was seen following antibiotic and α-terpineol treatment. The inhibition of Proteobacteria, which includes numerous recognized Gram-negative pathogens, further substantiates the antibacterial and microbiota-modulating effects of α-terpineol (22). The treatments elicited specific and significant changes at the genus level. The antibiotic and phytogenic treatments consistently diminished the prevalence of many clostridial groups, including Clostridia_UCG-014 and Clostridia_vadinBB60_group. The genus Clostridium exhibits significant diversity, with numerous species participating in protein fermentation pathways that may generate potentially toxic metabolites (50). A decrease in these groups may consequently improve the cecal metabolic profile. The most notable genus-level alteration was observed in the combination group (TEP2+CNI), which showed a significant increase in the Eubacterium coprostanoligenes group and a decline in the Rikenellaceae RC9 gut group. *Eubacterium* species are substantial producers of butyrate, a short-chain fatty acid that serves as the primary energy source for colonocytes, strengthens the gut barrier, and exhibits anti-inflammatory properties. The enhancement of this group with the combined treatment indicates a unique ecological impact of the two chemicals together, despite the systemic anti-inflammatory effect being less significant. This underscores the intricacy of microbial regulation, in which systemic and local (microbial) effects may not always align. The elevated levels of Cyanobacteria and Synergistota in groups administered α-terpineol, along with the rise of Campilobacterota in groups receiving α-terpineol (either alone or in combination), demonstrate that the influence of these phytogenics is not uniformly inhibitory but also facilitates the proliferation of specific bacterial taxa. The ecological functions of these taxa in the rabbit cecum remain inadequately characterized and may be neutral or context-dependent (51). Their proliferation may enhance the beta-diversity (between-sample variation) noted in the TEP2 and TEP2+CNI groups during the PLS-DA analysis, suggesting that α-terpineol, specifically, may augment the uniqueness or adaptability of the microbial community structure. Finally, our study demonstrates that the isolated monoterpenoids 1,8-cineole and α-terpineol from *C. longepaniculatum* exhibit excellent dual-target modulatory effects on intestinal health in meat rabbits. Their modes of action include both direct effects on the host, such as enhancing the intestinal barrier and reducing systemic inflammation, and indirect effects through the advantageous reconfiguration of the cecal microbiota. This encompasses augmenting microbial diversity, fostering beneficial Actinobacteriota, inhibiting inflammation-related Deferribacteria, and regulating specific species involved in gut metabolism and health. The discovery that individual supplementation at 80 mg/kg yielded more consistent systemic anti-inflammatory effects than the combined lower-dose treatment is essential for future product design. This indicates that the bioactivity of these chemicals is dependent on dosage and that their interaction is intricate, necessitating additional research to refine synergistic ratios. This research establishes a robust scientific basis for employing 1,8-cineole and α-terpineol as targeted, effective, and sustainable substitutes for in-feed antibiotics to enhance gut health and production in livestock.

It should be noted that α-terpineol and 1,8-cineole are also widely distributed in pine oil, eucalyptus oil, tea tree oil, and various herbs and spices. The essential oil of Eucalyptus kochilli subsp. Borealis contains approximately 97.32% 1,8-cineole (41). α-Terpineol is the main component (%, w/w) in the essential oils of marjoram (Origanum majorana, 73%), Pinus pinaster (67.3%), and clary sage (Salvia sclarea, 47.4%) (39). To date, the supply of 1,8-cineole has relied primarily on extraction and isolation from plants, while part of the α-terpineol supply is provided by chemical synthesis (39, 41). However, traditional methods such as extraction, isolation, and chemical synthesis suffer from low yields, high costs, and significant environmental pressure. In recent years, with technological advances, the biosynthetic pathways of monoterpenes have offered a green, efficient, and sustainable alternative for the production of essential oils, which is of great significance for achieving high-quality, stable, and sustainable supply as well as the development and application of the essential oil industry (52).

## 5 Conclusion

This study demonstrates that dietary supplementation with the pure monoterpenoids 1,8-cineole and α-terpineol, derived from *Cinnamomum longepaniculatum*, significantly enhances gut health in meat rabbits through separate yet complementary mechanisms. Supplementation at 80 mg/kg of each compound individually markedly enhanced intestinal barrier integrity, as evidenced by decreased serum concentrations of D-lactic acid and diamine oxidase, and mitigated systemic inflammation by downregulating critical proinflammatory cytokines, such as TNF-α and IL-1β. Simultaneously, both drugs enhanced cecal microbial ecology by augmenting overall diversity, promoting favorable Actinobacteriota, and reducing inflammation-associated taxa, such as Deferribacterota. This study reveals that concurrent administration of 1,8-cineole and α-terpineol, each at 40 mg/kg, produced a diminished systemic anti-inflammatory effect compared with individual supplementation at the full dosage. This suggests that the bioactivity of these chemicals may follow a non-linear, dose-dependent interaction paradigm, in which efficacy is maximized at specific concentrations rather than through a straightforward additive combination. Under the experimental conditions used, α-terpineol supplementation at 80 mg/kg demonstrated the greatest and most consistent benefits across both inflammatory and microbiological parameters, establishing it as a notably promising candidate for further study. These findings provide substantial scientific evidence for the use of specific phytogenic chemicals as targeted substitutes for in-feed antibiotics. Future studies must focus on elucidating the exact molecular processes underlying their distinct effects and interaction dynamics, as well as refining delivery formulations to enhance their stability and bioavailability in commercial feeding systems.

## Data Availability

The raw sequence data have been deposited in the Genome Sequence Archive (Genomics, Proteomics & Bioinformatics 2025) at the National Genomics Data Center (Nucleic Acids Res 2025), China National Center for Bioinformation / Beijing Institute of Genomics, Chinese Academy of Sciences, https://ngdc.cncb.ac.cn/gsa, accession number: CRA043851.
